# Cyclin G2 suppresses Wnt/β-catenin signaling and inhibits gastric cancer cell growth and migration through Dapper1

**DOI:** 10.1186/s13046-018-0973-2

**Published:** 2018-12-14

**Authors:** Jinlan Gao, Chenyang Zhao, Qi Liu, Xiaoyu Hou, Sen Li, Xuesha Xing, Chunhua Yang, Yang Luo

**Affiliations:** The Research Center for Medical Genomics, School of Life Sciences, China Medical University, No.77 Puhe Road, Shenyang North New Area, Shenyang, Liaoning Province 110122 People’s Republic of China

**Keywords:** Cyclin G2, Dpr1, Gastric Cancer, Tumor suppressor, Wnt/β-catenin signaling

## Abstract

**Background:**

Gastric cancer is one of the most common malignant tumors. Cyclin G2 has been shown to be associated with the development of multiple types of tumors, but its underlying mechanisms in gastric tumors is not well-understood. The aim of this study is to investigate the role and the underlying mechanisms of cyclin G2 on Wnt/β-catenin signaling in gastric cancer.

**Methods:**

Real-time PCR, immunohistochemistry and in silico assay were used to determine the expression of cyclin G2 in gastric cancer. TCGA datasets were used to evaluate the association between cyclin G2 expression and the prognostic landscape of gastric cancers. The effects of ectopic and endogenous cyclin G2 on the proliferation and migration of gastric cancer cells were assessed using the MTS assay, colony formation assay, cell cycle assay, wound healing assay and transwell assay. Moreover, a xenograft model and a metastasis model of nude mice was used to determine the influence of cyclin G2 on gastric tumor growth and migration in vivo. The effects of cyclin G2 expression on Wnt/β-catenin signaling were explored using a TOPFlash luciferase reporter assay, and the molecular mechanisms involved were investigated using immunoblots assay, yeast two-hybrid screening, immunoprecipitation and Duolink in situ PLA. *Ccng2*^*−/−*^ mice were generated to further confirm the inhibitory effect of cyclin G2 on Wnt/β-catenin signaling in vivo. Furthermore, GSK-3β inhibitors were utilized to explore the role of Wnt/β-catenin signaling in the suppression effect of cyclin G2 on gastric cancer cell proliferation and migration.

**Results:**

We found that cyclin G2 levels were decreased in gastric cancer tissues and were associated with tumor size, migration and poor differentiation status. Moreover, overexpression of cyclin G2 attenuated tumor growth and metastasis both in vitro and in vivo. Dpr1 was identified as a cyclin G2-interacting protein which was required for the cyclin G2-mediated inhibition of β-catenin expression. Mechanically, cyclin G2 impacted the activity of CKI to phosphorylate Dpr1, which has been proved to be a protein that acts as a suppressor of Wnt/β-catenin signaling when unphosphorylated. Furthermore, GSK-3β inhibitors abolished the cyclin G2-induced suppression of cell proliferation and migration.

**Conclusions:**

This study demonstrates that cyclin G2 suppresses Wnt/β-catenin signaling and inhibits gastric cancer cell growth and migration through Dapper1.

**Electronic supplementary material:**

The online version of this article (10.1186/s13046-018-0973-2) contains supplementary material, which is available to authorized users.

## Background

Gastric cancer is one of the most common malignant tumors and is the third leading cause of cancer deaths worldwide according to recent estimates [1, 2]. Although the precise mechanisms leading to gastric cancer are not fully understood, abnormal activation of the Wnt/β-catenin signaling has been suggested as the major risk factor contributing to cancer development [3, 4]. Therefore, identifying the suppressors of the Wnt/β-catenin signaling pathway may not only increase our knowledge of the pathobiology of this disease, but also help us to identify a potential target for its treatment.

Cyclin G2 is an unconventional cyclin because it plays a negative role in the progression of the cell cycle [5, 6]. It was reported that expression level of cyclin G2 was correlated with gastric cancer progression [7]. We previously showed that overexpression of cyclin G2 inhibited gastric cancer cell growth in liquid cultures and in soft agar [8]. Besides gastric cancer, the potential relationship between low cyclin G2 expression and tumor progression has also been previously reported in oral cancer, esophageal cancer, colorectal cancer, thyroid cancer, epithelial ovarian cancer and prostate cancer [9–15]. However, there are few reports on the identity of pathways and the precise mechanisms that mediate the roles of cyclin G2 in gastric tumorigenesis and other cancers.

Wnt/β-catenin signaling plays a pivotal role in the regulation of cell proliferation, differentiation, embryonic patterning, and tumorigenesis [16–18]. The key component of the Wnt/β-catenin signaling pathway is β-catenin. This protein is phosphorylated and degraded by a cytoplasmic complex comprising glycogen synthase kinase 3β (GSK-3β), axin, CK (casein kinase) Iα and adenomatous polyposis coli (APC) acting through the ubiquitin-proteasome pathway [19, 20]. Wnt ligands bind to cell surface receptor Frizzled (Fzd)/low-density lipoprotein-related protein (LRP) co-receptor complexes, which in turn activate Dishevelled (Dvl) [21, 22]. Dvl antagonizes the phosphorylation of β-catenin by inhibiting GSK-3β activity thereby triggering β-catenin stabilization [23–25]. The accumulated cytoplasmic β-catenin translocates to the nucleus and interacts with the T cell factor/lymphocyte-enhanced factor (TCF/LEF) to form a complex which functions as a transcriptional coactivator of the Wnt-target genes such as *c-MYC*, *CCND1*, and *RUNX2* [26, 27]. It was reported that β-catenin and APC gene mutations are involved in the Wnt-induced gastric cancers [4, 28]. In addition, other molecules have been found to contribute to the effects of Wnt/β-catenin signaling pathway in gastric cancer [29–31]. Several antagonists have been reported to play important roles in other biological functions mediated by Wnt/β-catenin signaling.

We previously reported that cyclin G2 inhibited osteogenesis through Wnt/β-catenin pathway [32], which also contributed to the development of gastric cancer. In this study, the role of cyclin G2 in gastric cancer in vitro and in vivo mediated by Wnt/β-catenin signaling was determined. Dapper1 (Dpr1) was identified as the target of the cyclin G2-induced inhibition on the Wnt/β-catenin signaling. This study demonstrates the inhibitory function of cyclin G2 in gastric cancer proliferation and migration through the Wnt/ β-catenin signaling and explored the underlying mechanisms.

## Methods

### Cell lines and cell culture

The human gastric cancer cell line (AGS), human cervical cell line (HeLa), human embryonic kidney cell line (HEK-283), a monkey kidney-derived cell line (COS-7) and a human colon cancer cell line (HT-29) were obtained from the American Type Culture Collection (Manassas, VA, USA). An immortalized human gastric epithelial mucosa cell line (GES-1), two gastric cancer cell lines (SGC-7901 and MGC-803) and the human colon cancer cell line (HT-29) were kept in our lab. SGC-7901, MGC-803 and AGS cells were cultured in RPMI-1640 (Gibco®, Grand Island, NY, USA). GES-1, HEK-283, COS-7 and HT-29 were cultured in Dulbecco’s Modified Eagle’s Medium (DMEM; Gibco®). All culture media were supplemented with 10% fetal bovine serum (FBS), penicillin and streptomycin and maintained at 5% CO_2_ at 37 °C.

### Human tissue samples

Forty-five pairs of human gastric cancer tissue samples and matched adjacent non-tumor tissues were obtained from patients who had undergone surgical resection at The First Hospital of China Medical University (CMU) between 2009 and 2010, and who were diagnosed with gastric cancer based on the histopathological evaluation. Matched, adjacent, non-tumor tissue was obtained from a portion of each resected specimen farthest from the tumor (> 5 cm). All samples were immediately frozen in liquid nitrogen after resection and stored at − 80 °C. No local or systemic treatments were performed on these patients prior to surgery. This study was approved by the Research Ethics Committee of CMU, Shenyang, China. Informed consent was obtained from all patients.

### In silico analysis

*CCNG2* mRNA levels were retriedved from Oncomine database [33] (http://www.oncomine.org). Five gastric sample datasets were assessed: Ooi Gastric [34]; DErrico Gastric [35]; Forster Gastric [36]; Chen Gastric [37]; Cho Gastric [38]. *CCNG2* negative and positive patients were categorized based on the median centered intensity values of *CCNG2* probes.

### Tissue microarray assay immunohistochemical assay

Glass slides with tissue microarrays containing 180 specimens from different stages of gastric carcinoma and paired adjacent non-tumor tissues were provided by Shanghai Outdo Biotech Co. (Shanghai, China). Immunohistochemical assay was used to determine cyclin G2 and Ki-67 expression as described previously [39]. Briefly, The slides were incubated with primary antibodies overnight at 4 °C followed by incubation with biotin secondary antibodies. All immunohistochemistry images were determined using Image-Pro plus 4.0 software (Media Cybernetics, Rockville, MD, USA). The measurement parameters included density mean, area sum, and integral optical intensity (IOD). The optical density was calibrated and the area of interest was set through: hue, 0–30; saturation, 0–255; intensity, 0–255. The image was converted to gray scale image, and then the values were counted. Researchers were blinded to each sample.

### Plasmid construction

The GFP-tagged human CKI δ plasmid was constructed by PCR and subcloned into pEGFP-N3. Using of the mammalian expression plasmid for 3 × FLAG-tagged cyclin G2 was described previously [32]. We also generated Dpr1-specific and cyclin G2-specific shRNA vectors by cloning double-stranded oligonucleotides targeting Dpr1 or cyclin G2 mRNA into a PGPU6/GFP/Neo vector (GenePharma; Shanghai, China) using *Bbs* I/*Bam* HI sites. Target sequences were described previously [23, 40], and the oligonucleotides used (for a successful Dpr1-specific shRNA) were: forward oligo, 5′-caccgatctgcagatctcataggatttcaagagaatcctatgagatctgcagatttttttg-3′, and reverse oligo, 5′-gatccaaaaaaatctgcagatctcataggattctcttgaaatcctatgagatctgcagatc-3′. For a successful cyclin G2-specific shRNA, a forward oligo, 5′-caccgtattccatccactcatgattcaagagatcatgagtggatggaatattttttg-3′, and reverse oligo 5′-gatccaaaaaatattccatccactcatgatctcttgaatcatgagtggatggaatac-3′ were used. These were annealed to form a fragment containing a 5’ *Bam* HI and a 3’ *Bbs* I restriction site overhang, and then cloned into a restriction enzyme-digested plasmid to create PGPU6/GFP/Neo-shDACT1 (shDACT1) and PGPU6/GFP/Neo-shG2 (shG2). PGPU6/GFP/Neo-shNC (shNC) was used as a negative control (GenePharma).

### Transient gene transfection, recombinant retrovirus and lentivirus generation and cell infection

Transient transfection was conducted using Lipofectamine™ 2000 (Invitrogen, Carlsbad, CA, USA) and jetPRIME (Polyplus Transfection, France) according to the manufacturer’s recommendations as described previously. Recombinant cyclin G2 retrovirus and the negative control retrovirus were packaged and used as described before [32]. FLAG-tagged cyclin G2 was cloned into a lentivirus-GFP lentiviral vector and generated by Genechem Co., Ltd.(Shanghai, China), and then it was used to infect cells according to the manufacturer’s instruction. GFP-lentiviral vector was used as a negative control.

### Reporter plasmid and dual luciferase assay

The TCF/LEF reporter plasmid (M50 Super 8× TOPFlash, plasmid #12456; Addgene, Cambridge, MA, USA) and the mutant TCF/LEF reporter plasmid (M51 Super 8× FOPFlash, plasmid #12457; Addgene) were gifts from Dr. Randall T. Moon [41]. The *Renilla* luciferase control plasmid was purchased from Promega (Madison, WI, USA). Cells were transfected with 8× TOPFlash or 8× FOPFlash and other additional plasmids as indicated along with pRL-TK (Promega) to normalize the transfection efficiencies. After transfection for 48 h, cells were washed with PBS and lysed in passive lysis buffer (Promega). The lysates were subjected to a dual luciferase assay system (Promega) and processed according to the manufacturer’s protocol, followed by measurement using a Lumat LB-9501 luminometer (Berthold Analytical Instruments, Nashua, NH, USA). TOP/FOP ratios represented the mean of three independent experiments, performed in triplicate.

### RNA isolation, reverse transcription and real-time polymerase chain reaction

Total RNA was isolated from cultured cells using TRIzol® reagent (Invitrogen) according to the manufacturer’s instructions. After quantification of RNA samples, cDNA was synthesized from 1 μg of total RNA using a TaKaRa RNA polymerase chain reaction (PCR) Kit (AMV) version 3.0 (TaKaRa, Dalian, China), and subjected to a real-time PCR reaction using SYBR® Premix Ex Taq™ (TaKaRa) according to the manufacture’s instruction. PCR primer sequences for the amplification of human *GAPDH* (Primer Bank ID 7669492a2, forward, 5’-TGTTGCCATCAATGACCCCTT-3′, and reverse, 5’-CTCCACGACGTACTCAGCG-3′), human *CCNG2* (Primer Bank ID 4757936a1, forward, 5’-TGCCTAGCCGAGTATTCTTCT-3′, and reverse, 5’-TGTTTGTGCCACTTTGAAGTTG-3′), were obtained from the Harvard Medical School Primer Bank database (http://pga.mgh.harvard.edu/primerbank/), a public resource for PCR primers [42]. Human *GAPDH* was used as an internal control.

### Protein extraction, immunoprecipitation, and western blotting

Cells were lysed using a radio-immunoprecipitation assay (RIPA) buffer containing a Protease Inhibitor Cocktail and PhosSTOP Phosphatase Inhibitor Cocktail (both from Roche, Basel, Switzerland) as previously described [43]. For immunoprecipitation, cell lysates were incubated with appropriate antibodies and protein G-Sepharose and then subjected to western blotting. The membranes were blotted with appropriate primary antibodies followed by horseradish peroxidase-coupled secondary antibodies and then visualized using chemiluminescence (DNR Bio-Imaging Systems, Jerusalem, Israel). For western blot analysis, β-tubulin was used as a loading control. Antibodies were obtained from various sources, including Sigma–Aldrich (St. Louis, MO, USA; anti-β-tubulin, anti-β-catenin, anti-phosphoserine/threonine, anti-Flag M2 and anti-CCNG2), Cell Signaling Technology (Beverley, MA, USA; anti-p–β-catenin ser33, ser37 and thr42), Abcam (Cambridge, UK; Anti-Phosphoserine, anti-CCNG2 and anti-DACT1), Affinity Biosciences (Changzhou, China; anti-CCNG2), Wanleibio (Shenyang, China; anti-GAPDH), Proteintech Group (Chicago, IL, USA, anti-CCNG2) and Santa Cruz Biotechnology (Santa Cruz, CA, USA; anti-Dvl2, anti-GSK-3β, anti-cyclin G2, anti-DACT1, anti-p-GSK-3β-Ser9 and anti-c-Myc).

### Cell proliferation assay

Cells were infected with cyclin G2 recombinant lentivirus for 24 h as indicated, followed by counting and seeding in 96-well plates at a density of 5 × 10^3^ cells per well, in a total volume of 100 μL per well. Cell proliferation was assessed at the indicated times using the MTS assay (Promega) according to the manufacturer’s instructions. Briefly, 10 μL of MTS solution was added to each well and cells were incubated for another 2 h at 37 °C. The relative cell viability was determined by scanning with an ELISA reader with a 450 nm filter. Experiments were performed in triplicate for each group.

### Cell-cycle analysis

The cells were harvested and washed with PBS, and then fixed overnight with cold 70% ethanol at − 20 °C. Cellular DNA was stained with cell cycle staining solution containing propidium iodide (PI) (BD, San Diego, USA) at 4 °C in dark. Cell cycle was determined using a FACS Calibur and analyzed with the Cell Quest acquisition software (BD Biosciences).

### Colony formation assay

The cells were placed into a 6-well plate (1000 cells/well) and cultured for 14 days. The cell colonies were fixed with methanol, and then stained with 0.5% Crystal violet. Colonies were observed using a microscope and colonies with > 50 cells/colony were counted.

### Wound healing assays

Cells were grown overnight in a 24-well plates (4 × 10^5^ per well) until reaching confluency, and the confluent cell monolayers were manually wounded using a micropipette tip under an angel of around 30 degrees to scrap the cells to perform wound healing assays. Wound size was verified under a microscope to ensure that all wounds were the same width, and the cell migration rate in the cell-free area was monitored over the indicated times using light microscopy. These experiments were repeated three times for each group.

### Transwell® migration assays

Transwell® migration assays were performed in 24-well fitted plates with 8 μm pore size membranes (Corning Inc., Lowell, MA, USA) as previously described. Briefly, 2 × 10^5^ serum-starved cells were plated on Transwell® inserts supplemented with serum-free medium. Lower chambers were filled with complete medium and then incubated for 24 h. Cells on the upper surface of membranes were completely removed with a cotton stick and the migrated cells were fixed with 4% paraformaldehyde (PFA), followed by the Crystal violet staining or the Giemsa stain. The 10 randomly chosen fields of the stained cells were counted to determine cell migration indices using a light microscope. Experiments were performed three times in triplicate.

### Yeast two-hybrid screening

Wild-type cyclin G2 cDNA was first cloned into a pGBKT7 plasmid and used as a bait to screen the human fetal brain cDNA library (both from Clontech/TaKaRa). Interacting clones were grown on a selection medium that lacked the amino acids His, Leu and Trp. Auxotrophic marker genes and the β-galactosidase assay were used to screen for positive clones. Plasmids of positive clones were isolated, amplified and sequenced. Interactions were further confirmed by retransforming the identified plasmids together with the bait. The identified DNA sequences were further characterized by BLAST analysis on the NCBI database (http://blast.ncbi.nlm.nih.gov/Blast.cgi) to determine the identity of potential interacting proteins for cyclin G2.

### Duolink in situ proximity ligation assay

The Duolink in situ proximity ligation assay (PLA) was performed according to the manufacturer’s protocol (Sigma–Aldrich). In brief, COS-7 cells and SGC-7901 cells were plated on glass coverslips, rinsed three times with PBS and fixed in 4% formaldehyde in PBS for 10 min. The cells were permeabilized in 0.5% Triton X-100 for 5 min and blocked with 3% BSA in PBS for 60 min at 37 °C. After blocking, cells were then incubated with antibodies against cyclin G2 and Dpr1 in PBS containing 1% BSA overnight at 4 °C, followed by incubation with corresponding secondary antibodies conjugated with PLA probes for 60 min at 37 °C in the dark. Cells were washed three times in PBS. Duolink and DAPI signals were detected using a confocal microscopy.

### Xenograft tumor assay and metastasis nude mice assay

Eighteen 4-week-old female BALB/c nude mice were randomly divided into three groups, each containing 6 mice. Nude mice were subcutaneously injected with 2 × 10^6^ SGC-7901 cells (blank), SGC-7901 cells overexpressing cyclin G2 or control GFP (NC) in 0.2 ml PBS into the upper back region of nude mice. The tumors were measured every 3 days after sizeable tumors formation by caliper measurement of the subcutaneous tumor mass. The tumor volume was calculated using the following formula: V = (Width^2^ × Length)/2. Inoculated mice were sacrificed on day 25 and the tumors were excised for analysis.

In metastasis nude mice assay, twelve 4-week-old female BALB/c nude mice were randomly divided into two groups, each containing 6 mice. 10^6^ cells of SGC-7901 cells expressing ectopic cyclin G2 or GFP in 0.1 ml PBS were injected into nude mice through the lateral tail vein. The mice were sacrificed and dissected after 4 weeks of injection. The metastatic foci in lung tissue were counted in slides with H&E staining. All animal experiments were performed in accordance with the accepted standards of the Ethics Committee of CMU.

### Mice and MEF cell

The TALEN-targeted *Ccng2* knockout (KO) mice (*Ccng2*^*−/−*^) of the C57BL/6 genetic background were generated by Cyagen (Cyagen Biosciences, Guangzhou, China). Wild type (*WT*) C57BL/6 mice were used as the control in our study. All animal experiments were performed in accordance with the accepted standards of the Ethics Committee of CMU. *Ccng2* WT and *Ccng2*^*−/−*^ primary MEFs were obtained from embryos derived from cyclin G2 KO C57BL/6 mice heterozygote breeding at 13.5 days post coitum according to standard procedure. MEFs were cultured in DMEM supplemented with penicillin (100 mg/ml) and streptomycin (100 mg/ml) and used at early passages.

### Statistical analyses

Data are presented as the mean ± standard deviation (SD) and statistically analyzed using Student’s *t*-test (two-sided) or one-way ANOVA. Tukey’s post hoc comparison was used to determine the statistical differences between treatment groups. Differences were considered statistically significant if *P* < 0.05.

## Results

### Expression levels of cyclin G2 were downregulated and associated with proliferation, migration and differentiation in gastric cancer

**Fig. 1:**
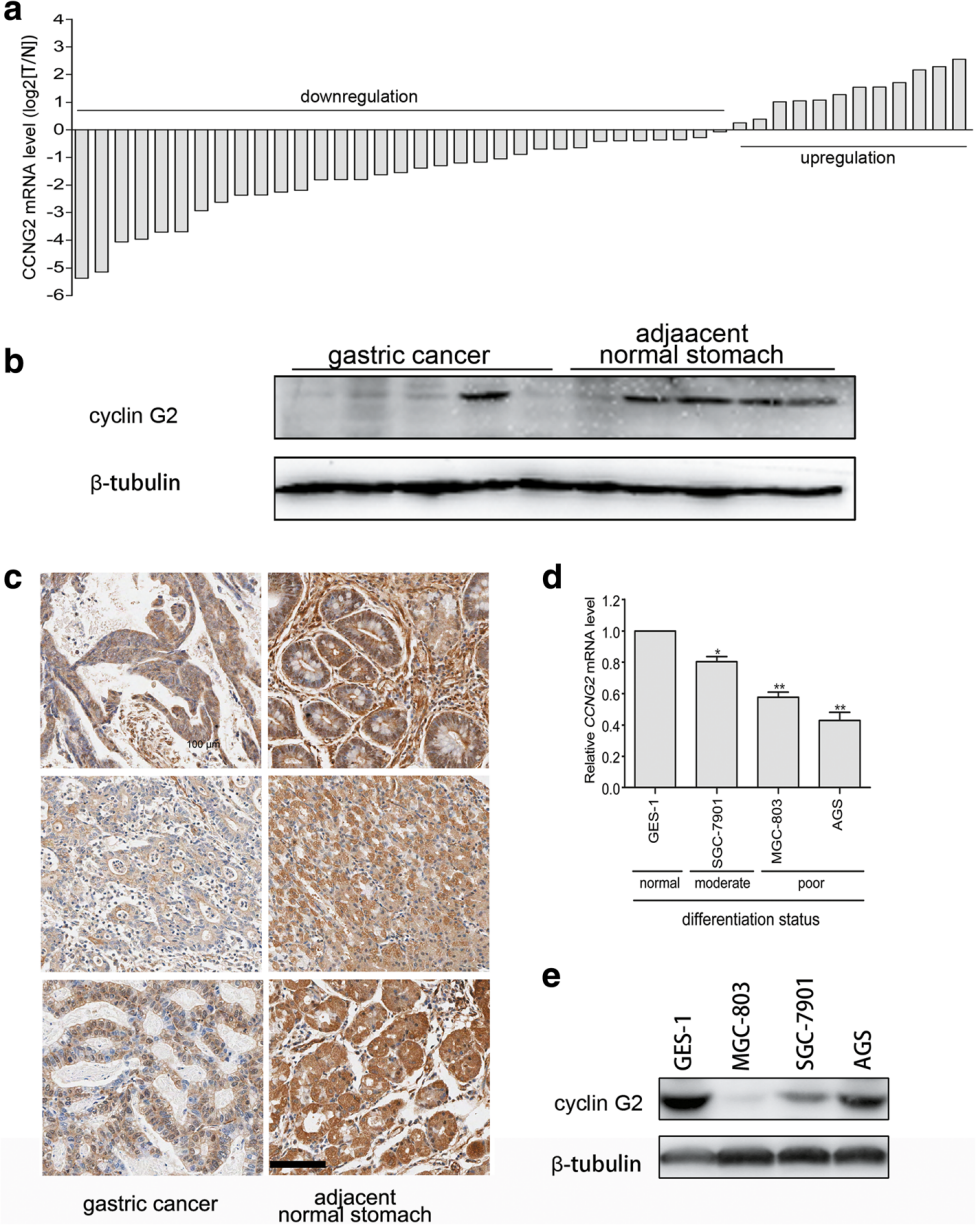
Cyclin G2 expression is down-regulated in gastric cancer. **a** Relative mRNA expression of *CCNG2* in 45 paired gastric cancer and adjacent normal tissues. The quantification of *CCNG2* mRNA was performed using qRT-PCR. Data are presented as log_2_ values in gastric cancer tissues relative to matched adjacent non-tumor tissues. **b** Western blot analysis for the cyclin G2 protein levels in ten gastric cancer tissues and adjacent non-cancerous gastric tissues. **c** Representative immunohistochemical staining with cyclin G2 antibody in gastric cancer and paired adjacent normal tissues. Six representative cases are shown. Scale bar = 100 μm. **d** Relative mRNA expression of *CCNG2* in GES-1 and three gastric cancer cell lines (SGC-7901, AGS and MGC-803) with different differentiation statuses. *CCNG2* mRNA was quantified by qRT-PCR. Data from gastric cancer cell lines are expressed relative to the GES-1 *CCNG2* mRNA level (a normal gastric epithelial cell line that was chosen as a control). Results are presented as the means of values. Bars indicate standard deviation (SD; *n* = 3). *P < 0.05; **P < 0.01. **e** Comparison of the protein expression levels of cyclin G2 in three gastric cell lines and human normal gastric epithelial cell line

To study the expression patterns of cyclin G2 in gastric cancer, mRNA expression levels of cyclin G2 were quantitated by real-time PCR in 45 pairs of gastric cancer and matched adjacent normal gastric tissue samples. Down-regulation of cyclin G2 mRNA was observed in 33 of the 45 gastric cancer samples compared with paired normal tissues (Fig. 1a). Low level of cyclin G2 expression was also shown in immunoblotting assay in gastric cancer compared with normal gastric tissues (Fig. 1b). Furthermore, immunohistochemical staining of 90 gastric cancer and matched normal tissues revealed that protein levels of cyclin G2 were down-regulated in gastric cancer tissues (Fig. 1c). Moreover, the decreased expression of cyclin G2 was significantly associated with high tumor size (*P* < 0.01), poor tumor differentiation (*P* < 0.05) as well as metastasis status (*P* < 0.05; Table 1). Expression levels of cyclin G2 mRNA were also correlated to the differentiation status of gastric cancer cell lines, with the lowest mRNA cyclin G2 expression occurring in the poorly differentiated AGS cells and the highest cyclin G2 expression occurring in the well-differentiated immortalized human gastric epithelial mucosa cell line GES-1 (Fig. 1d). However, the protein level of cyclin G2 was not consistent with its mRNA expression in AGS cells (Fig. 1e). These findings suggested that cyclin G2 was involved in the progression of gastric cancers.

**Table 1:** Correlation between expression cyclin G2 and clinical Features in Gastric cancer

Clinicopathologic features	n	cyclin G2 expression		x^2^	*P* value
		Up-regulate or no change	Down-regulate		
Age(years)					
60 or younger	40	12	28	0.2209	0.6384
Older than 60	49	17	32		
Gender					
Male	67	21	46	0.1900	0.6629
Female	22	8	14		
Tumor size (cm3)					
≤ 5	8	6	2	7.1991	0.0073
> 5	81	23	58		
Depth of tumor invasion					
T1-T2	23	9	14	0.6912	0.4057
T3-T4	64	19	45		
Histologic type					
Well and moderate	27	13	14	4.2739	0.0387
Poor and undifferentiated	62	16	46		
TNM stage					
I	15	7	8	7.1151	0.0683
II	26	8	18		
III	38	8	30		
IV	10	6	4		
Metastasis status					
No	79	23	56	3.8547	0.0496
Yes	10	6	4		

NOTE. Cyclin G2 expression was up-regulated or unchanged in 29 gastric cancer tissues, down-regulated in 60 gastric cancer samples compared with the paired adjacent normal tissues. Both depth of tumor invasion and TNM stage: according to 2010 TNM classification of malignant tumors by the International Union Against Cancer. The statistical significance of the individuals was determined with *x*^*2*^ test

In addition, in silico assay was performed to determine the expression of cyclin G2 in gastric cancer using the bioinformatics tool Oncomine (www.oncomine.org) from five different datasets (334 cases) (Additional file 1 Figure S1). From Forster gastric (43 sample) datasets, we found no statistical differences between normal and gastric cancers. However, in DErrico gastric (69 sample) and Ooi gastric (200 cases) datasets, cyclin G2 expression was decreased in gastric cancers. In contrast, Chen gastric (132 samples) and Cho gastric (90 samples) datasets showed an increased cyclin G2 expression in gastric cancers.

### Cyclin G2 negatively regulated proliferation and migration of gastric cancer cells in vivo and in vitro

**Fig. 2:**
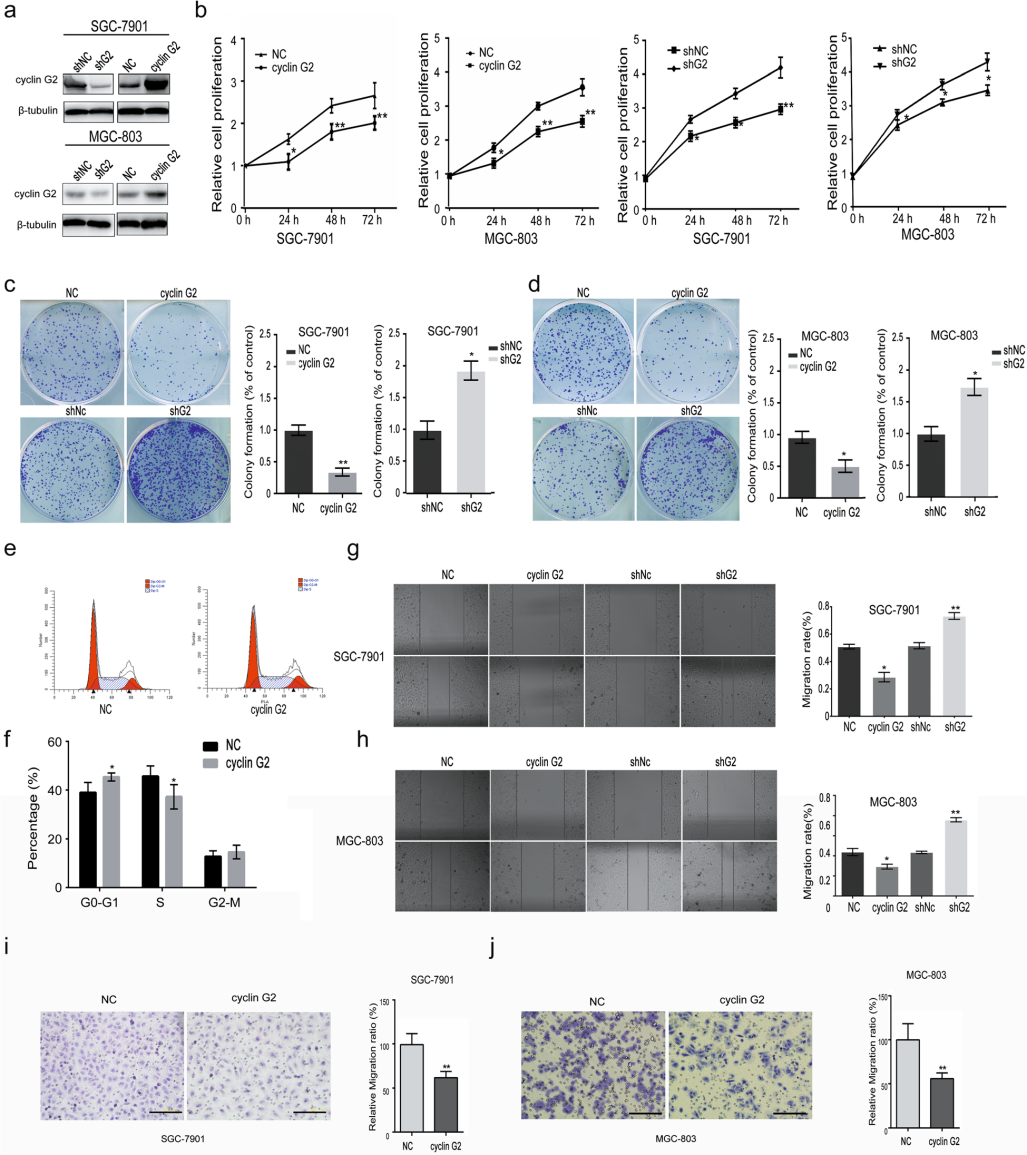
Cyclin G2 inhibits the proliferation and migration of SGC-7901 or MGC-803 gastric cancer cells. **a** Western blot analysis for the cyclin G2 protein levels in cyclin G2 overexpression and knockdown SGC-7901 or MGC-803 cells. **b** MTS cell proliferation assay in cyclin G2 overexpression and cyclin G2 knockdown SGC-7901 or MGC-803 cells (n = 3).*P < 0.05; **P < 0.01. **c** and **d** Colony formation assay in cyclin G2 overexpression and cyclin G2 knockdown SGC-7901 or MGC-803 cells (n = 3).*P < 0.05; **P < 0.01. **e** and **f** Representative images and quantification of cell cycle analysis after cyclin G2 overexpression in SGC-7901 cells by flow cytometry. **g** and **h** Representative images and quantification of the wound healing assay in cyclin G2 overexpression and cyclin G2 knockdown SGC-7901 or MGC-803 cells (n = 3).*P < 0.05; **P < 0.01. **i** and **j** Representative Transwell® migration assays in cyclin G2 overexpression SGC-7901 or MGC-803 cells determined by stained with Giemsa. The migration ratio is expressed as a percentage of the control cells at the indicated time-points (n = 3). **P < 0.01

To determine the effects of cyclin G2 on gastric cancer cells, the expression of cyclin G2 was induced by recombinant cyclin G2 lentivirus or down-regulated by shRNA plasmid in SGC-7901 and MGC-803 cells. The overexpression and knockdown of cyclin G2 were confirmed using western blot (Fig. 2a). The MTS assay and colony formation assays revealed that cell proliferation was inhibited in cyclin G2 overexpressed SGC-7901 and MGC-803 gastric cancer cells, but it was increased in cyclin G2 knockdown cells (Fig. 2b-d). Besides, ectopic expression of cyclin G2 resulted in cell cycle arrest at G1/S phase (Fig. 2e and f). In addition, the wound healing assay and Transwell^®^ migration assays revealed an inhibitory role of cyclin G2 on the migration of gastric cancer cells as shown in Fig. 2g-j. These findings are consistent with in vivo results showing that the expression of cyclin G2 was inversely correlated with tumor metastasis in gastric cancer samples.

**Fig. 3:**
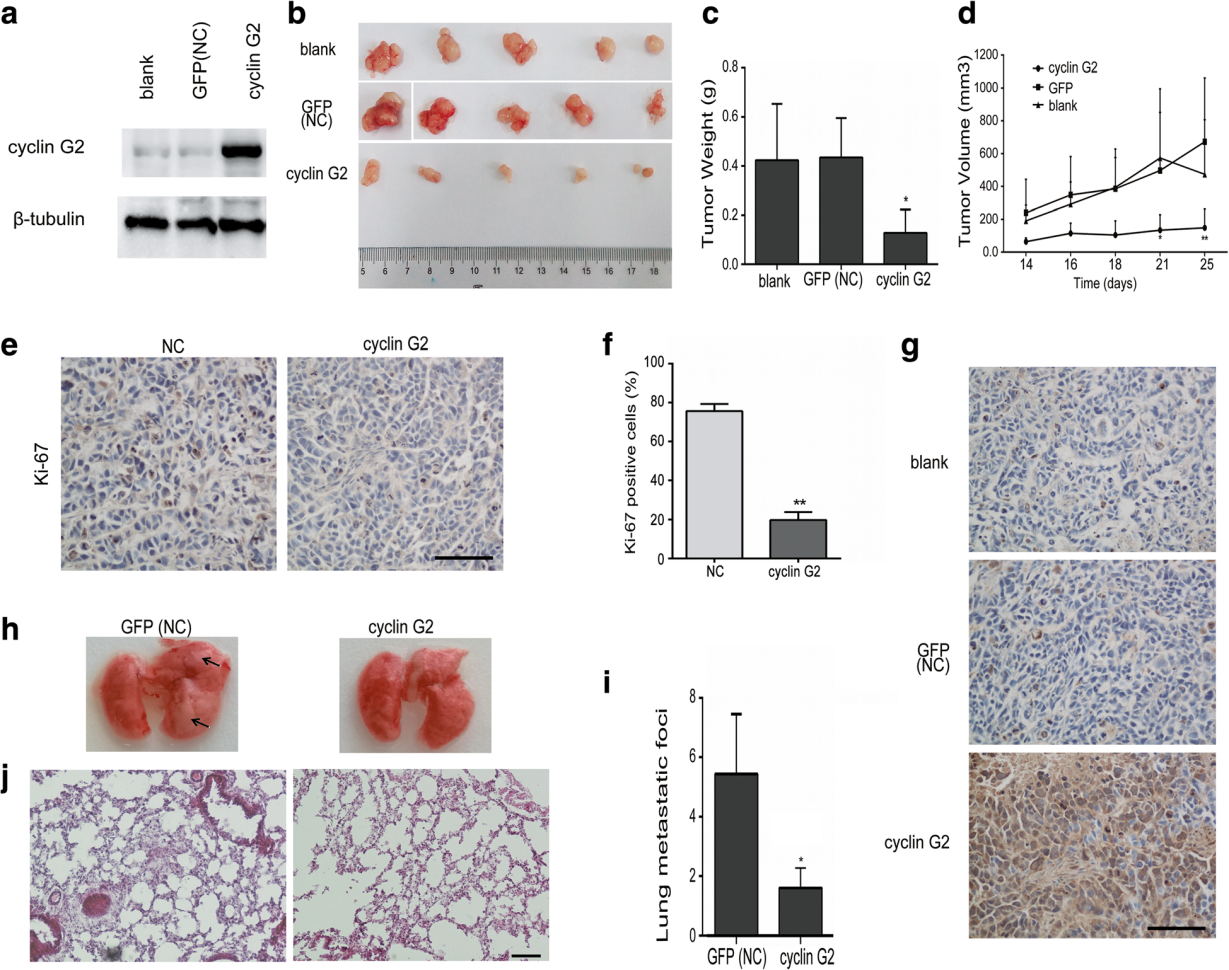
Cyclin G2 inhibited tumor growth and metastasis in vivo. **a** Western blot analysis for the cyclin G2 expression in SGC-7901 cells (blank), SGC-7901 cells overexpressing cyclin G2 and GFP (NC). **b–d** Cyclin G2 inhibited tumor growth in the xenograft nude mice. Nude mice were subcutaneously injected with SGC-7901 cells (blank), SGC-7901 cells overexpressing cyclin G2 or control GFP (NC) by lentivirus infection. Tumor sizes were measured after sizeable tumors formation (day 14). Inoculated mice were sacrificed on day 25 and the tumors were excised for analysis (*n* = 5). **b** Macroscopic appearance of the isolated tumor. **c** The tumor weights in cyclin G2-overexpressing, control vector GFP-overexpressing (NC) and untransfected SGC-7901 cell (blank) groups were compared (n = 5). *P < 0.05. **d** Comparison of tumor volume curves in uninfected SGC-7901 cells (blank), SGC-7901 cells overexpressing cyclin G2 and GFP (NC) groups in xenograft nude mice (n = 5). **e** Representative immunohistochemical staining with a Ki-67 antibody of the xenograft tumor from nude mice injected with SGC-7901 negative control cells and SGC-7901 cells overexpressing cyclin G2. Bar = 100 μm. **f** Graph represents percentage of Ki-67-positive cells out of the total cells in each field (n = 3). **P < 0.01. **g** Representative immunohistochemical staining of xenograft tumors from nude mice with cyclin G2 antibody. Bar = 100 μm. **h–i** The effect of cyclin G2 on gastric cancer metastasis in vivo. (h) Representative images of whole lungs from nude mice inoculated with SGC-7901 cells overexpressing cyclin G2 or control GFP (NC) by lentivirus infection. (i) Statistical analysis showed a decrease in the metastatic foci of the nude mice inoculated with SGC-7901 cells overexpressing cyclin G2 compared to those overexpressing GFP. *P < 0.05. j Representative H&E stain images of Lung metastasis. Bar = 100 μm

Moreover, a xenograft model in nude mice was used to determine the effect of cyclin G2 on gastric tumor growth in vivo. Overexpression of cyclin G2 remarkably suppressed tumor growth, as reflected by tumor volume and weight, compared with the blank group (without any transfection) and GFP vector control (NC) group cells (Fig. 3a-g). These results are in line with in vitro results. The decrease in cell proliferation caused by cyclin G2 in the xenograft model was also validated by immunohistochemical staining of Ki-67. Fewer proliferating cells were found in cyclin G2 overexpressing tumors (*P* < 0.01; Fig. 3e and f). To further investigate the inhibitory effect of cyclin G2 on metastasis in vivo, SGC-7901 cells expression ectopic cyclin G2 or GFP was injected into nude mice through the lateral tail vein. Metastatic foci in lungs showing in the control group. In contrast, few metastatic tumors were detected in mice injected with SGC-7901 cells overexpressing cyclin G2 (Fig. 3h-j). Taken together, cyclin G2 negatively regulated the proliferation and migration of gastric cancer cells in vivo and in vitro*.*

### Cyclin G2 negatively regulated Wnt/β-catenin signaling in vivo and in vitro

**Fig. 4:**
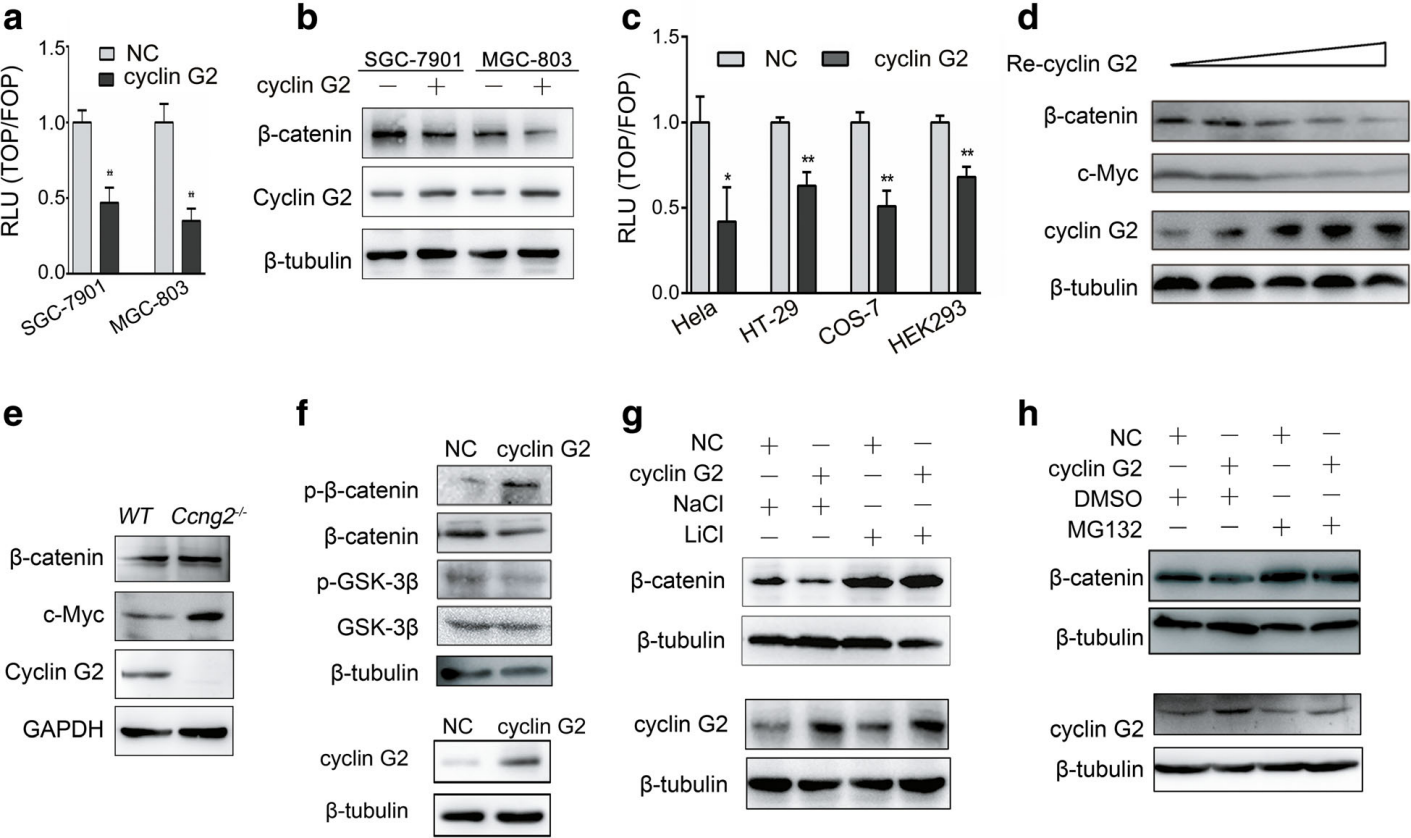
Cyclin G2 inhibits Wnt/β-catenin signaling by promoting β-catenin degradation in a GSK-3β dependent manner. **a** β-catenin transcriptional activity of SGC-7901 and MGC-803 cells overexpressing cyclin G2 or control vector (NC) was determined by a TOPFlash luciferase reporter assay (n = 3). *P < 0.05. **b** Western blot analysis for the β-catenin expression in ectopic cyclin G2 overexpression in SGC-7901 or MGC-803 cells compared with the negative vector control. **c** TOPFlash luciferase reporter assays showed that ectopic cyclin G2 suppressed β-catenin transcriptional activity in HeLa, HT-29, HEK-293, and COS-7 cells. **d** COS-7 cells infected with incremental concentrations of recombinant retroviruses encoding *CCNG2* (Re-cyclin G2) or a negative control gene were used for western blotting after 48 h incubation. Western blot analysis showed the overexpression of cyclin G2 inhibited β-catenin and c-Myc expression in a dose-dependent manner. **e** β-catenin and c-Myc expression levels in *Ccng2*^*−/−*^ and WT MEFs were analyzed by western blot. **f** Western blot analysis revealed that the expression and phosphorylation of β-catenin and GSK-3β in the negative control and cyclin G2-overexpressing COS-7 cells. **g** Western blot analysis for β-catenin protein levels in the control or cyclin G2-overexpressing COS-7 cells treated with or without GSK-3β inhibitor, LiCl (20 mM), for 24 h. NaCl was used as a negative control. **h** Western blot analysis to determine the β-catenin protein levels in the control or cyclin G2-overexpressing COS-7 cells treated with or without proteasome inhibitor, MG132 (25 μM), for 6 h. DMSO was used as a negative control

Based on the findings above, we explored the underlying molecular mechanisms through which cyclin G2 reduced the progression of gastric cancers. In our previous study, we found that cyclin G2 inhibited estrogen-induced osteogenesis through the Wnt/β-catenin pathway [32], which has been shown to play a pivotal role in embryogenesis and tumorigenesis [17, 44]. Therefore, we first determined whether cyclin G2 could inhibit the activity of TOPFlash reporter in gastric cancer cells. As shown in Fig. 4a, overexpression of cyclin G2 inhibited the β-catenin/TCF-mediated transcription of SGC-7901 and MGC-803 cells, suggesting that cyclin G2 acted as a negative regulator of the Wnt/β-catenin signaling in gastric cancer cells. We further examined the effects of cyclin G2 on protein levels of total β-catenin, which is a key component of the Wnt/β-catenin signaling pathway. Overexpression of cyclin G2 downregulated the expression of endogenous β-catenin both in SGC-7901 and MGC-803 cells (Fig. 4b). Similar effects of cyclin G2 on the Wnt/β-catenin signaling activity were also observed in HeLa, HT-29, COS-7 and HEK293 cells as confirmed by the TOPFLASH assays (Fig. 4c). Besides, COS-7 cells infected with incremental recombinant retrovirus encoding the *hCCNG2* gene showed that cyclin G2 inhibited the expression of β-catenin protein in a dose-dependent manner (Fig. 4d). To confirm the inhibitory effect of endogenous cyclin G2 on Wnt/β-catenin signaling in vivo, we generated cyclin G2 knockout mice (*Ccng2*^*−/−*^). Similarly, β-catenin protein level was upregulated in cyclin G2-deficient mouse embryonic fibroblasts (MEFs) compared with wild-type cells, indicating that endogenous cyclin G2 decreases β-catenin expression (Fig. 4e). Taken together, cyclin G2 regulates the Wnt/β-catenin signaling in a negative manner both in vitro and in vivo*.*

### Cyclin G2 promoted GSK-3β-induced β-catenin degradation

We further investigated the precise mechanisms through which cyclin G2 inhibited the Wnt/β-catenin signaling. Since β-catenin is primed by GSK-3β for ubiquitination and proteasomal degradation, we next assessed whether GSK-3β was involved in the cyclin G2-induced suppression of the β-catenin expression. No significant alteration in GSK-3β expression was found in cells overexpressing cyclin G2. However, overexpression of cyclin G2 notably reduced the expression of P-Ser9 GSK-3β, the inactive form of GSK-3β, and increased the phosphorylation of β-catenin (Fig. 4f). In addition, the ability of cyclin G2 to promote β-catenin degradation was compromised when GSK-3β activity was inhibited by LiCl (Fig. 4g). These results demonstrated that the role of cyclin G2 in promoting β-catenin degradation required the induction of GSK-3β activity. Moreover, treatment of cyclin G2-overexpressing cells with MG132, a proteasome inhibitor that inhibits the degradation of ubiquitin-conjugated proteins in mammalian cells, did not change the β-catenin protein levels while β-catenin expression was decreased by cyclin G2 in dimethyl sulfoxide (DMSO) treated group (Fig. 4h). These results indicated that cyclin G2 promoted β-catenin degradation through the ubiquitin-dependent proteolysis system. Taken together, cyclin G2 suppressed the Wnt/β-catenin activity by promoting GSK-3β-induced β-catenin degradation.

### Cyclin G2 interacted with Dpr1 to promote β-catenin degradation

**Fig. 5:**
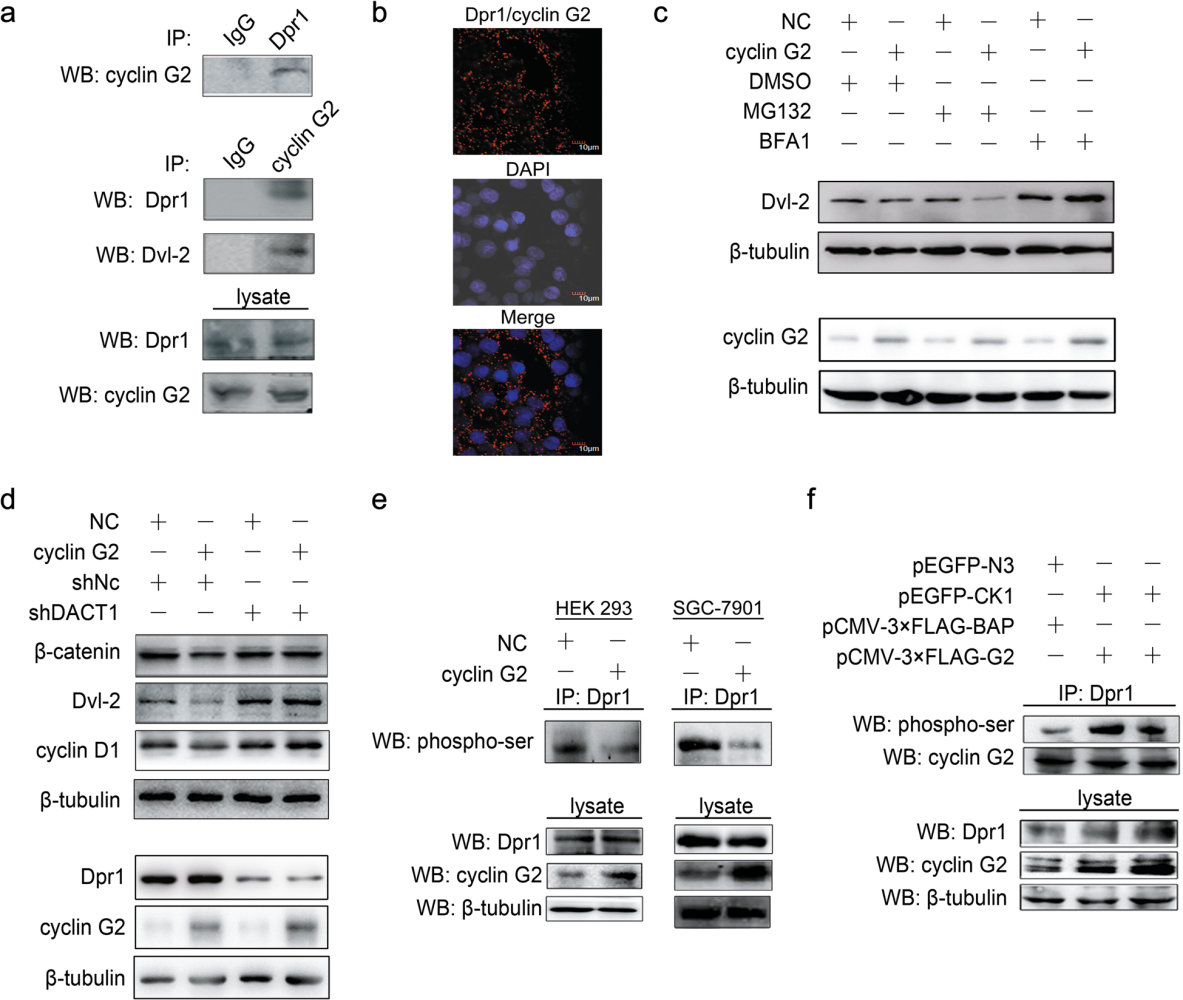
Cyclin G2 inhibits Wnt/β-catenin signaling through DPR1. **a** Cell lysates from COS-7 cells were immunoprecipitated with Dpr1 or IgG control antibody, followed by western blot analysis with a cyclin G2 antibody (upper panel). Total cellular protein from COS-7 cells was immunoprecipitated with an anti-cyclin G2 or IgG control antibody, then blotted with anti-DPR1 and anti-Dvl2 antibody, respectively (middle panel). Western blot analysis of endogenous Dpr1 and cyclin G2 protein level in COS-7 cells (lower panel). **b** Direct interaction of Dpr1 and cyclin G2 was detected using Duolink in situ PLA. Red spots represent the interaction of Dpr1 and cyclin G2. The nuclei were stained using DAPI and are shown in blue. Images were acquired using a confocal microscopy with a 40× objective. **c** Western blot analysis of the Dvl2 protein levels in the control or cyclin G2-overexpressing COS-7 cells treated with 25 μM MG132, 1 μM bafilomycin A1 (BFA1), or DMSO as a negative vehicle control for 6 h followed. **d** Overexpression of cyclin G2 down-regulated β-catenin and Dvl2 protein expression levels in COS-7 cells, whereas knockdown of Dpr1 attenuated the effect of cyclin G2. cyclin G2 overexpressing vector or control vector (NC) was co-transfected with Dpr1-specific (shDACT1) or nonspecific shRNA vectors (shNC) into COS-7 cells for 48 h followed by western blot analysis. **e** The phosphorylation level of Dpr1 was suppressed by cyclin G2. Cell lysates from HEK-293 and SGC-7901 cells overexpressing cyclin G2 or GFP as a negative control was immunoprecipitated with anti-Dpr1 antibodies, then immunoblotted with anti-Phosphoserine/threonine antibody (upper panel). Western blot analysis of Dpr1 and cyclin G2 expression in total cellular protein (lower panel). **f** Cyclin G2 inhibited the CKI-induced phosphorylation of Dpr1. HEK-293 cells were transfected with expression vectors encoding CKI (pEGFP-CKI) and cyclin G2 (pCMV-3 × FLAG-G2) or transfected with negative control vectors (pCMV-3 × FLAG-BAP or pEGFP-N3) for 48 h. The cell lysates were immunoprecipitated with Dpr1 antibodies and immunoblotted with anti-Phosphoserine antibody (upper panel). Dpr1 and cyclin G2 expression in total cellular protein was analysed by western blot (lower panel)

To identify the target that is necessary for protein that mediates the cyclin G2-induced inhibition of the Wnt/β-catenin signaling, we screened for cyclin G2-interacting proteins. We used wild-type cyclin G2 as a yeast two-hybrid bait to screen the human fetal brain library. One of the positive clones encoded full-length hDpr1, an inhibitor of the Wnt/β-catenin signaling pathway. The interaction between cyclin G2 and Dpr1 at endogenous levels in HEK-293 cells was confirmed using the co-immunoprecipitation assay (Fig. 5a). Direct interaction between cyclin G2 and Dpr1 was determined using the Duolink in situ PLA in fixed COS-7 cells (Fig. 5b). Besides, Dvl2 protein, which is suppressed by Dpr1 protein that serves as a negative regulator for both canonical and noncanonical Wnt signaling, was also detected in the same cyclin G2 immunoprecipitant (Fig. 5a). It was previously reported that Dpr1 could inhibit the Wnt/β-catenin signaling pathway by promoting Dvl2 degradation [24, 25]. We, therefore, determined whether cyclin G2 influenced the stability of Dvl2 protein. COS-7 cells overexpressing cyclin G2 were treated with or without different inhibitors of protein degradation. Overexpression of cyclin G2 decreased the protein levels of Dvl2, but the proteasome inhibitor, MG132, reversed this effect while the lysosome inhibitor, bafilomycin A1, did not have any obvious effect (Fig. 5c).

The Dvl2 degradation caused by Dpr1 led to the inhibition of Wnt/β-catenin signaling [25]. Thus, we assessed whether Dpr1 was required for cyclin G2 to inhibit Wnt/β-catenin signaling. A cyclin G2-overexpressing or negative control vector was co-transfected with Dpr1-specific or nonspecific shRNA vectors into COS-7 cells. The endogenous Dpr1 protein level was inhibited by Dpr1-specific shRNA, and the downregulation of β-catenin by cyclin G2 overexpression was abolished in Dpr1 knockdown cells (Fig. 5d). A similar expression pattern of Dvl2 and cyclin D1 proteins were also observed, further confirming that the suppression of Wnt/β-catenin signaling by cyclin G2 required Dpr1 protein.

Yet to this point, the mechanism by which cyclin G2 modulated Dpr1 to influence Wnt/β-catenin signaling was not clear. Dpr1 was reported to inhibit Wnt/β-catenin signaling when unphosphorylated, but it promoted Wnt/β-catenin signaling when phosphorylated by casein kinase (CK) Iδ/ε [45]. We, therefore, determined whether the phosphorylation level of Dpr1 was influenced by cyclin G2. As shown in Fig. 5e, the results indicated that the phosphorylation level of Dpr1 was down-regulated when cyclin G2 was overexpressed in HEK-293 cells (Fig. 5e left panel). Similar results were obtained in SGC-7901 gastric cancer cells(Fig. 5e right panel). Moreover, cyclin G2 inhibited the ability of CKI to phosphorylate Dpr1 (Fig. 5f). The evidence presented in this section suggests that cyclin G2 interacts and impact Dpr1 phosphorylation, thereby inhibiting Wnt/β-catenin signaling.

### Cyclin G2 inhibited gastric cancer proliferation and migration through Wnt/β-catenin signaling

**Fig. 6:**
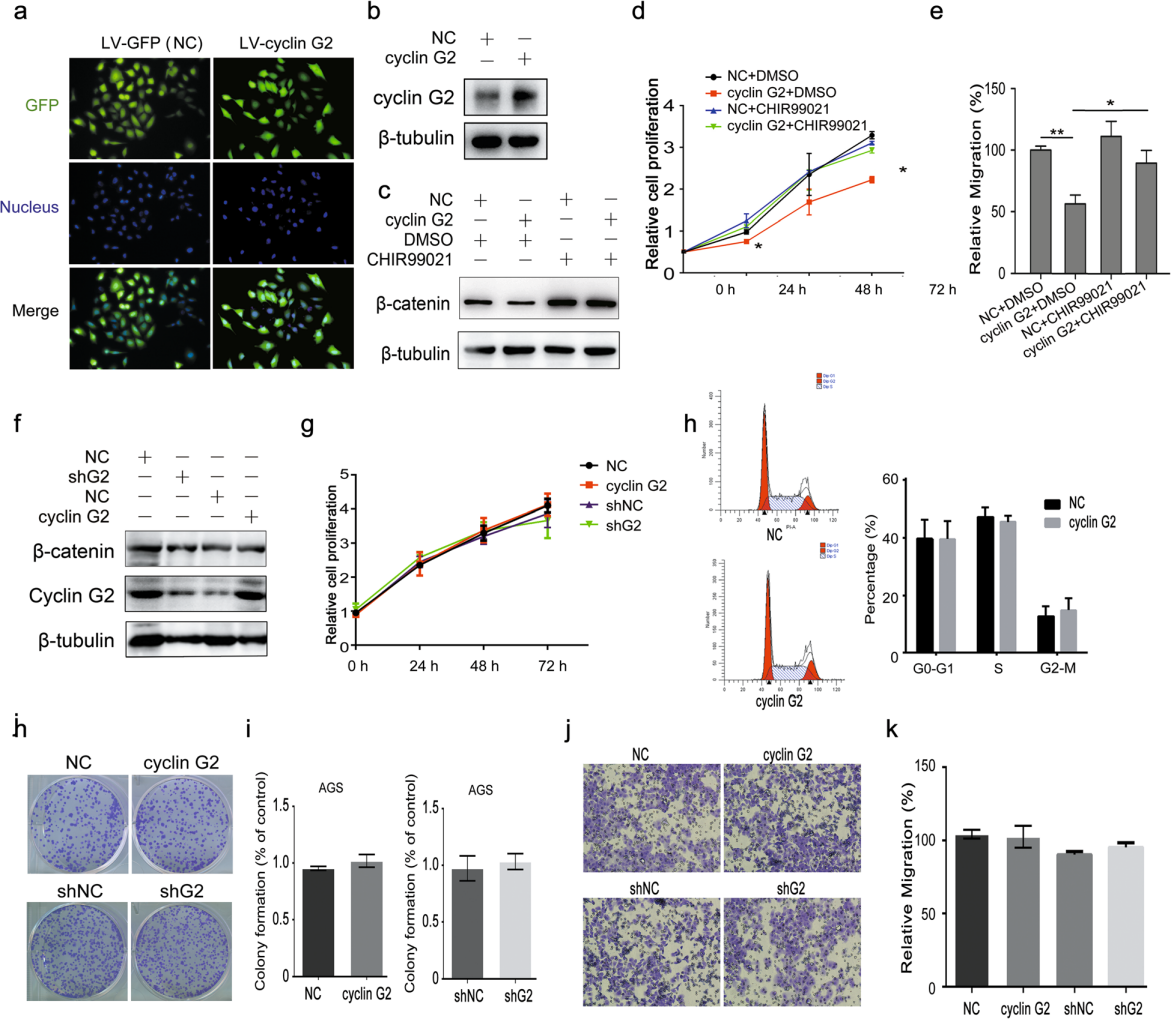
Cyclin G2 inhibits the gastric cancer proliferation and migration hrough Wnt/β-catenin signaling. **a-e** SGC-7901 cells were infected with cyclin G2 recombinant lentiviruses (LV-cyclin G2) or negative control lentivirus (LV-GPF) for 42 h and then treated with CHIR99021 or DMSO as a negative vehicle control. **a** and **b** The infection efficiency of recombinant lentivirus was confirmed by measurement of GFP vector expression (Original magnification, × 200) and western blot analysis of cyclin G2. **c** Cell lysates were subjected to immunoblot analysis to measure β-catenin protein expression level. **d** The cell proliferation of SGC-7901 cells was measured by the MTS assay (*n* = 6). *P < 0.05; **P < 0.01. **e** The cell migration of SGC-7901 cells was measured by Transwell^®^ migration assays. The data were presented as migrated cells as a percentage of the controls cells at the indicated time-points (*n* = 3). *P < 0.05; **P < 0.01. **f-k** AGS cells were overexpressed or knockdown of cyclin G2. **f** Cell lysates were subjected to immunoblot analysis to determine β-catenin protein expression level in cyclin G2 overexpression or knockdown AGS cells compared with negative control. **g** MTS cell proliferation assay, (**i and j**) colony formation assay and (**k and l**) Transwell® migration assay in cyclin G2 overexpression or knockdown AGS cells compared with the negative control (n = 3). **h** Cell cycle distributions were detected by flow cytometry analysis in cyclin G2 overexpression AGS cells compared with the negative control (n = 3). *P < 0.05; **P < 0.01

To explore the contribution of Wnt/β-catenin signaling to the observed cyclin G2-induced inhibition of gastric cancer proliferation and migration, SGC-7901 cells overexpressing cyclin G2 or control gene were generated by recombinant lentivirus infection assays. The infection efficiency was confirmed using a fluorescence microscope and western blot (Fig. 6a and b). CHIR99021, a highly selective inhibitor of GSK3β was used to activate Wnt/β-catenin signaling [46]. As expected, CHIR99021 abolished the decrease in β-catenin expression caused by cyclin G2 compared to vehicle controls (Fig. 6c). Cell proliferation and migration were assessed by MTS and Transwell^®^ migration assays, respectively. Consistent with the β-catenin protein level, CHIR99021 attenuated the cyclin G2-induced suppression of cell proliferation and migration of SGC-7901 gastric cancer cells (Fig. 6d and e). In addition, we overexpressed or knockdown cyclin G2 in AGS cells which have a missense mutation of glycine to glutamic acid at codon 34 in β-catenin NH2 terminus and altered potential GSK-3β phosphorylation sites to interfere with GSK-3β-mediated β-catenin degradation [47]. No obvious differences of β-catenin expression, cell proliferation, cell cycle, colony formation and cell migration were observed among cyclin G2 overexpression or knockdown AGS cells and negative control (Fig. 6f-k). Taken together, we can conclude that cyclin G2 inhibits gastric cancer proliferation and migration and suppresses Wnt/β-catenin signaling through Dpr1.

## Discussion

In the present study, we show that cyclin G2 was downregulated in clinical gastric cancer tissue and exerted inhibitory effects on proliferation and migration of gastric cancer cells in vitro and in vivo. Moreover, we showed a relationship between cyclin G2 and β-catenin, the key component of the Wnt/β-catenin signaling, which suggested that cyclin G2 negatively regulate this pathway in gastric cancer. Overexpression of cyclin G2 increased the degradation of β-catenin, and inhibited the expression of β-catenin downstream target genes, indicating that the cyclin G2 had a negative effect on the β-catenin function. Using GSK-3β inhibitors and a GSK-3β-mediated β-catenin phosphorylation site mutation AGS cell line, we revealed that cyclin G2 inhibited gastric cancer proliferation and migration through the Wnt/β-catenin signaling. Most importantly, Dpr1 was identified as the target for cyclin G2 to inhibit Wnt/β-catenin signaling. Taken together, this work reveals the cyclin G2 to suppressss the Wnt/β-catenin Signaling and inhibits gastric cancer cell growth and migration through Dapper1.

It has been reported that cyclin G2 is abnormally expressed in many types of cancers and is an apoptosis regulator, but its mechanisms in cancer progression remain elusive [9–15]. In the present study, we first determined the expression of cyclin G2 in gastric cancer tissues and cell lines. Low mRNA and protein expression of cyclin G2 in gastric cancer indicated that cyclin G2 is involved in the progression of gastric cancers (Fig. 1). This result is in accordance with previous studies in which low expression of cyclin G2 was observed in gastric cancers compared with normal gastric tissue. We also checked 5 online datasets(334 cases), the mRNA expression profiles of cyclin G2 among five oncomine datasets are not consistent (Additional file 1 Figure S1). Previously, we reported that cyclin G2 inhibited the osteogenesis of murine myoblast C2C12 cells through the canonical Wnt signaling [32]; this suggested that cyclin G2 may play a role in other types of cells, including cancer cells. Indeed, another study demonstrated that cyclin G2 inhibits epithelial-to-mesenchymal transition by disrupting the Wnt/β-catenin signaling in epithelial ovarian cancer [48]. Our findings that cyclin G2 suppressed β-catenin expression and TOP/FOP luciferase activities in gastric cancer cells, as well as in HeLa, HT-29, HEK293 and COS-7 cells (Fig. 4a and c), strongly provide evidence that cyclin G2 acts as a suppressor of Wnt/β-catenin signaling. Additionally, Chir99021 abolished the cyclin G2-induced inhibition on SGC-7901 cells proliferation and migration in vitro (Fig. 6d and e), suggesting that cyclin G2 inhibited gastric cancer through the Wnt/β-catenin signaling. Besides SGC-7901 and MGC-803 cell lines, we performed a series of experiments to determine the effect of overexpressing cyclin G2 in AGS cells, which is a commonly used gastric cancer cell line, using cell proliferation assay, colony formation assay and transwell assay. However, there were no obvious differences between cyclin G2-overexpressing cells and the negative control AGS cells(Fig. 6g and k). It is reported that AGS cell line containing a missense mutation of glycine to glutamic acid at codon 34 in β-catenin NH2 terminus altering its potential phosphorylation by GSK-3β [47]. The different effects of cyclin G2 between SGC-7901, MGC-803 and AGS cell lines indicated that cyclin G2 inhibited gastric cancer tumor growth and migration through the Wnt/β-catenin signaling in a GSK3β-mediated manner, although the precise molecular mechanisms remain to be elucidated.

β-catenin phosphorylation influences the stability of β-catenin and the functions of Wnt/β-catenin signaling [20]. We therefore tested whether cyclin G2 suppressed β-catenin expression by decreasing its stability. The observation that cyclin G2 induced the phosphorylation and the subsequent ubiquitin-dependent degradation of β-catenin implies that cyclin G2 may target GSK-3β to inhibit β-catenin stability (Fig. 4f). This hypothesis was confirmed in the subsequent experiments using GSK-3β inhibitors.

**Fig. 7:**
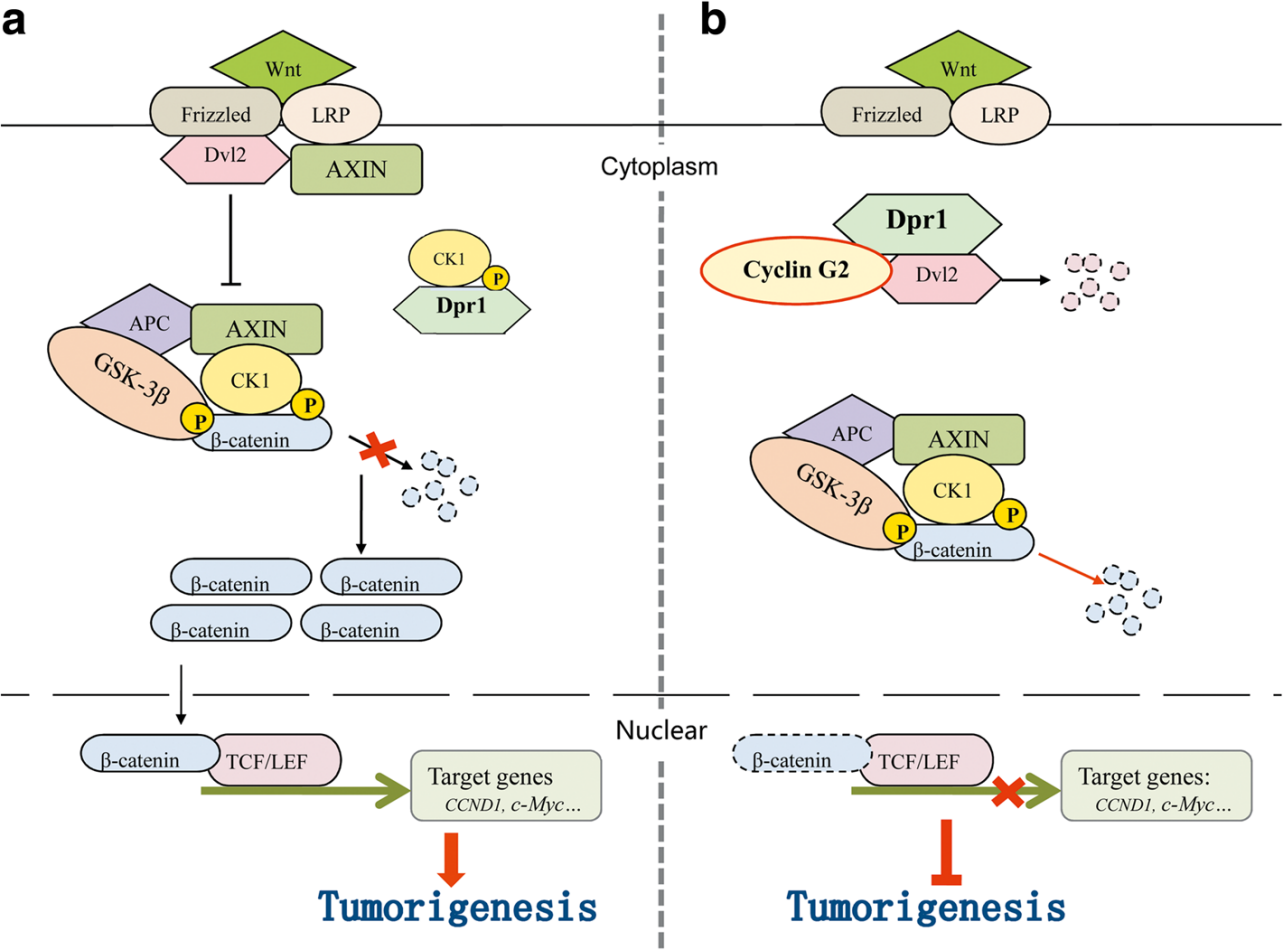
Hypothesied pathway showing the inhibitory mechanism of cyclin G2 on Wnt/β-catenin signaling through Dpr1. **a** Wnt ligands bind to Fzd and LRP co-receptor complexes, which in turn activates Dvl2. Dvl2 inhibits the activity of GSK-3β triggering β-catenin phosphorylation and preventing its degradation. Stabilized β-catenin activates gene expression of the Wnt/β-catenin signaling pathway and upregulates tumorigenesis. The inhibitory effect of Dpr1 on the Wnt/β-catenin signaling is blocked when phosphorylated by CKI. **b** Cyclin G2 interacts with Dpr1 and impacts its phosphorylation level, enhancing proteasome-dependent degradation of Dvl2. As a result, increasing the ability of GSK-3β to phosphorylate β-catenin and accelerate its degradation. This leads to suppression of Wnt/β-catenin signaling and inhibition of tumorigenesis of gastric cancer

However, to this stage, we still did not know how cyclin G2 regulates GSK-3β to inhibit the β-catenin stability. Using yeast two-hybrid screening, several proteins that interact with cyclin G2, including hDpr1, were identified. Dpr1 is a member of a conserved family of Dvl2-binding proteins that promote Dvl2 degradation [23]. We found the endogenous interaction among Dpr1, Dvl2 and cyclin G2 in the same protein complex (Fig. 5a). Furthermore, Dvl2 expression was downregulated by cyclin G2 overexpression (Fig. 5c). Dpr1 was reported to inhibit Wnt/β-catenin signaling when unphosphorylated, but promoted Wnt/β-catenin signaling when phosphorylated by casein kinase Iδ/ε [45]. The most interesting finding was that cyclin G2 inhibited the ability of CKI to phosphorylate Dpr1 (Fig. 5f). Besides, Dpr1-specific shRNA-mediated knockdown of Dpr1 expression decreased the inhibitory effect of cyclin G2 on the induction of β-catenin protein degradation (Fig. 5d). Collectively, our results suggested that cyclin G2 suppresses the Wnt/β-catenin signaling and inhibits gastric cancer cell growth and migration through Dapper1 (Fig. 7).

In summary, this study established that cyclin G2 suppresses Wnt/β-catenin signaling in gastric cancer, acting through association with and impact Dpr1 phosphorylation to stimulate β-catenin degradation in a GSK-3β-dependent manner. Our findings suggest a new mechanism of cyclin G2 in gastric cancer development. Thus, further investigation of the biological function of cyclin G2 may provide a molecular basis for the development of candidate therapeutic targets for gastric cancer.

## Conclusions

Taken together, these findings reveal that cyclin G2 suppresses Wnt/β-catenin signaling and inhibits gastric cancer cell growth and migration through Dapper1. Our findings suggest a new mechanism of cyclin G2 in gastric cancer development. Thus, cyclin G2 might be a candidate therapeutic target for gastric cancer treatment.

## Additional files

Additional file 1: **Figure S1.** In silico assay of cyclin G2 expression level in gastric cancer from Oncomine. **Figure S2.** The prognostic value of Cyclin G2 in gastric cancer. **Figure S3.** Positive and negative controls of immunohistochemistry assay. Bone marrow sections was used to validate cyclin G2 and Ki-67 antibody. Positive immunostaining presented as brown color counterstained with haematoxylin. IgGs against the species where the primary antibody was produced were used as negative controls of the staining (IgG). (ZIP 1323 kb)

## Abbreviations

APC: Adenomatous polyposis coli
BFA1: Bafilomycin A1
CK: Casein kinase
CMU: China Medical University
DMEM: Dulbecco’s Modified Eagle’s Medium
DMSO: Dimethyl sulfoxide
Dpr1: Dapper1
Dvl: Dishevelled
FBS: Fetal bovine serum
Fzd: Frizzled
GSK-3β: Glycogen synthase kinase 3β
IOD: Integral optical intensity
KO: Knockout
LEF: Lymphocyte-enhanced factor
LRP: Low-density lipoprotein-related protein
PFA: Paraformaldehyde
PI: Propidium iodide
PLA: Proximity ligation assay
SD: Standard deviation
shDACT1: PGPU6/GFP/Neo-shDACT1
shG2: PGPU6/GFP/Neo-shG2
TCF: T cell factor
WT: Wild-type
