# Supplementary material for: Cyclin G2 suppresses Wnt/β-catenin signaling and inhibits gastric cancer cell growth and migration through Dapper1
Source: J Exp Clin Cancer Res. 2018 Dec 14;37:317. doi: 10.1186/s13046-018-0973-2 (PMC6295076; doi:10.1186/s13046-018-0973-2)
Supplement: Supplementary file 1 — Figure S1. In silico assay of cyclin G2 expression level in gastric cancer from Oncomine. Figure S2. The prognostic value of Cyclin G2 in gastric cancer. Figure S3. Positive and negative controls of immunohistochemistry assay. Bone marrow sections was used to validate cyclin G2 and Ki-67 antibody. Positive immunostaining presented as brown color counterstained with haematoxylin. IgGs against the species where the primary antibody was produced were used as negative controls of the staining (IgG). (ZIP 1323 kb) [file 13046_2018_973_MOESM1_ESM.zip › 1105 Supplemetal Materials.docx]

Supplemetal Materials

**Methods**

*In silico* analysis

*CCNG2* mRNA levels were retriedved from Oncomine database (Rhodes et al. 2007; http://www.oncomine.org). Five gastric sample datasets were assessed: [Ooi (Ooi et al. 2009)](https://www.oncomine.org/resource/ui/component/dataset.html?component=d:146939097);  [[DErrico](https://www.oncomine.org/resource/ui/component/dataset.html?component=d:146939077) ([DErrico](https://www.oncomine.org/resource/ui/component/dataset.html?component=d:146939077) et al. 2009)](https://www.oncomine.org/resource/ui/component/dataset.html?component=d:146939097); [[Forster](https://www.oncomine.org/resource/ui/component/dataset.html?component=d:156636606) ([Forster](https://www.oncomine.org/resource/ui/component/dataset.html?component=d:156636606) et al. 2011)](https://www.oncomine.org/resource/ui/component/dataset.html?component=d:146939097);  [[Chen](https://www.oncomine.org/resource/ui/component/dataset.html?component=d:169) ([Chen](https://www.oncomine.org/resource/ui/component/dataset.html?component=d:169)et al. 2003)](https://www.oncomine.org/resource/ui/component/dataset.html?component=d:146939097);  [[Cho](https://www.oncomine.org/resource/ui/component/dataset.html?component=d:156636652) ([Cho](https://www.oncomine.org/resource/ui/component/dataset.html?component=d:156636652)et al. 2011)](https://www.oncomine.org/resource/ui/component/dataset.html?component=d:146939097). *CCNG2* negative and positive patients were categorized based on the median centered intensity values of *CCNG2* probes. The prognostic value of Cyclin G2 in gastric cancer was investigated in oncolnc using TCGA database.

**Result**

*In silico* assay was performed to determine the expression of cyclin G2 in gastric cancer using the bioinformatics tool Oncomine (www.oncomine.org) from five different datasets (334 cases) (Supplenmetal Fig1). From [Fonster](https://www.oncomine.org/resource/ui/component/dataset.html?component=d:146939077) (43 sample) datasets, we found no statistical differences between normal and gastric cancers. However, in [DErrico](https://www.oncomine.org/resource/ui/component/dataset.html?component=d:146939077) (69 sample) and Ooi (200 cases) datasets, cyclin G2 expression was decreased in gastric cancers (Figure 2a). In contrast, Chen (132 samples) and Cho (90 samples) datasets showed an increased cyclin G2 expression in gastric cancers. The association between cyclin G2 expression and the survival rate was also assessed using online TCGA data (www.oncolnc.org), and the results didn't show a specific association between cyclin G2 expression and a high survival rate (Supplenmetal Figure 2).

**Figure legend**

**Supplemental figure 1.** *In silico* assay of cyclin G2 expression level in gastric cancer from Oncomine

**Supplemental figure 2.** The prognostic value of Cyclin G2 in gastric cancer

**Supplemental figure 3.** Positive and negative controls of immunohistochemistry assay

Mouse bone marrow sections was used to validate cyclin G2 and Ki-67 antibody. Positive immunostaining presented as brown color counterstained with haematoxylin. IgGs against the species where the primary antibody was produced were used as negative controls of the staining (IgG).
