# Supplementary figures and images for: Cyclin G2 suppresses Wnt/β-catenin signaling and inhibits gastric cancer cell growth and migration through Dapper1
Source: J Exp Clin Cancer Res. 2018 Dec 14;37:317. doi: 10.1186/s13046-018-0973-2 (PMC6295076; doi:10.1186/s13046-018-0973-2)

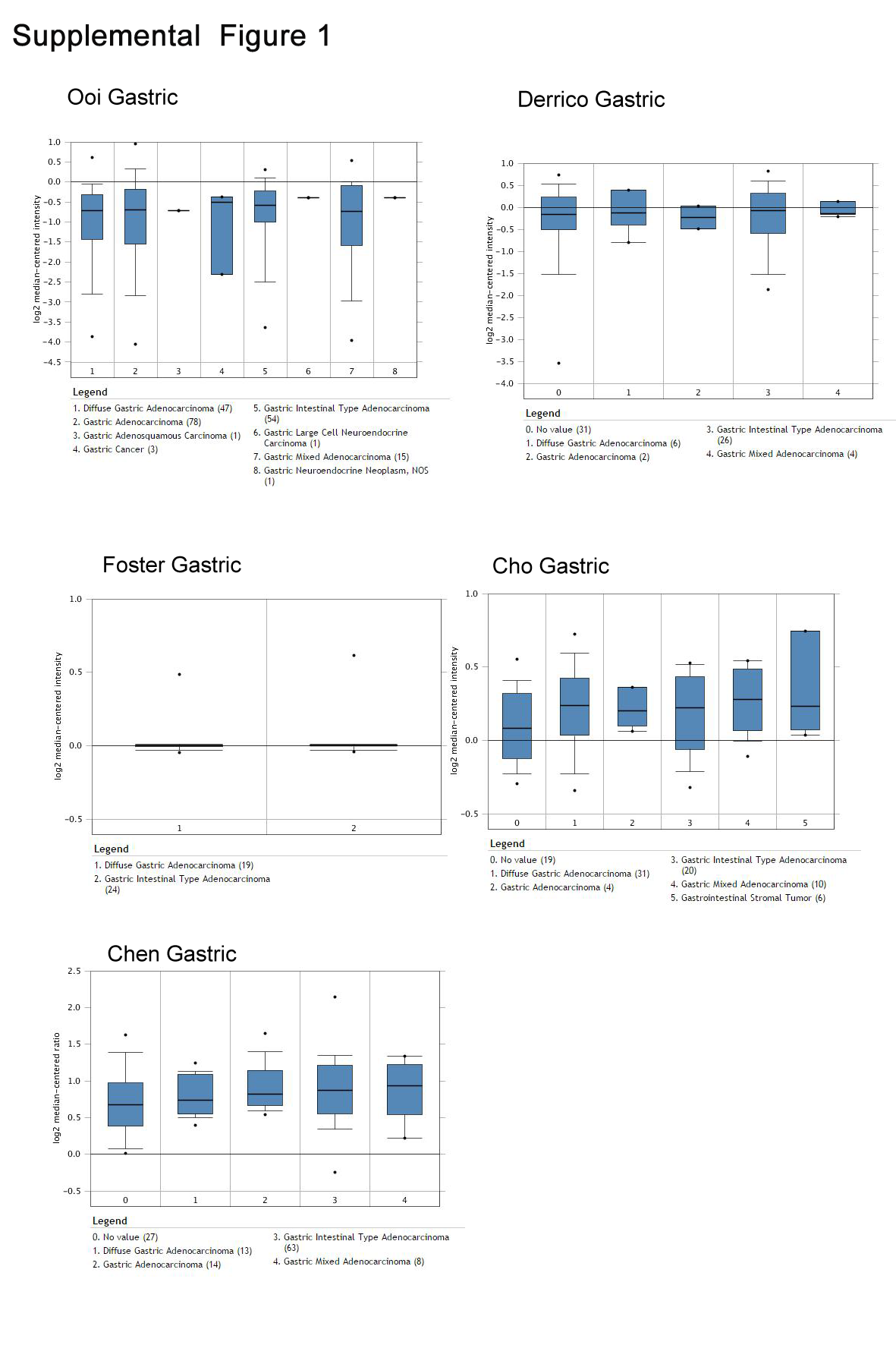

Supplement: Supplementary file 1 — Figure S1. In silico assay of cyclin G2 expression level in gastric cancer from Oncomine. Figure S2. The prognostic value of Cyclin G2 in gastric cancer. Figure S3. Positive and negative controls of immunohistochemistry assay. Bone marrow sections was used to validate cyclin G2 and Ki-67 antibody. Positive immunostaining presented as brown color counterstained with haematoxylin. IgGs against the species where the primary antibody was produced were used as negative controls of the staining (IgG). (ZIP 1323 kb) [file 13046_2018_973_MOESM1_ESM.zip › supplemental figure1.tif]

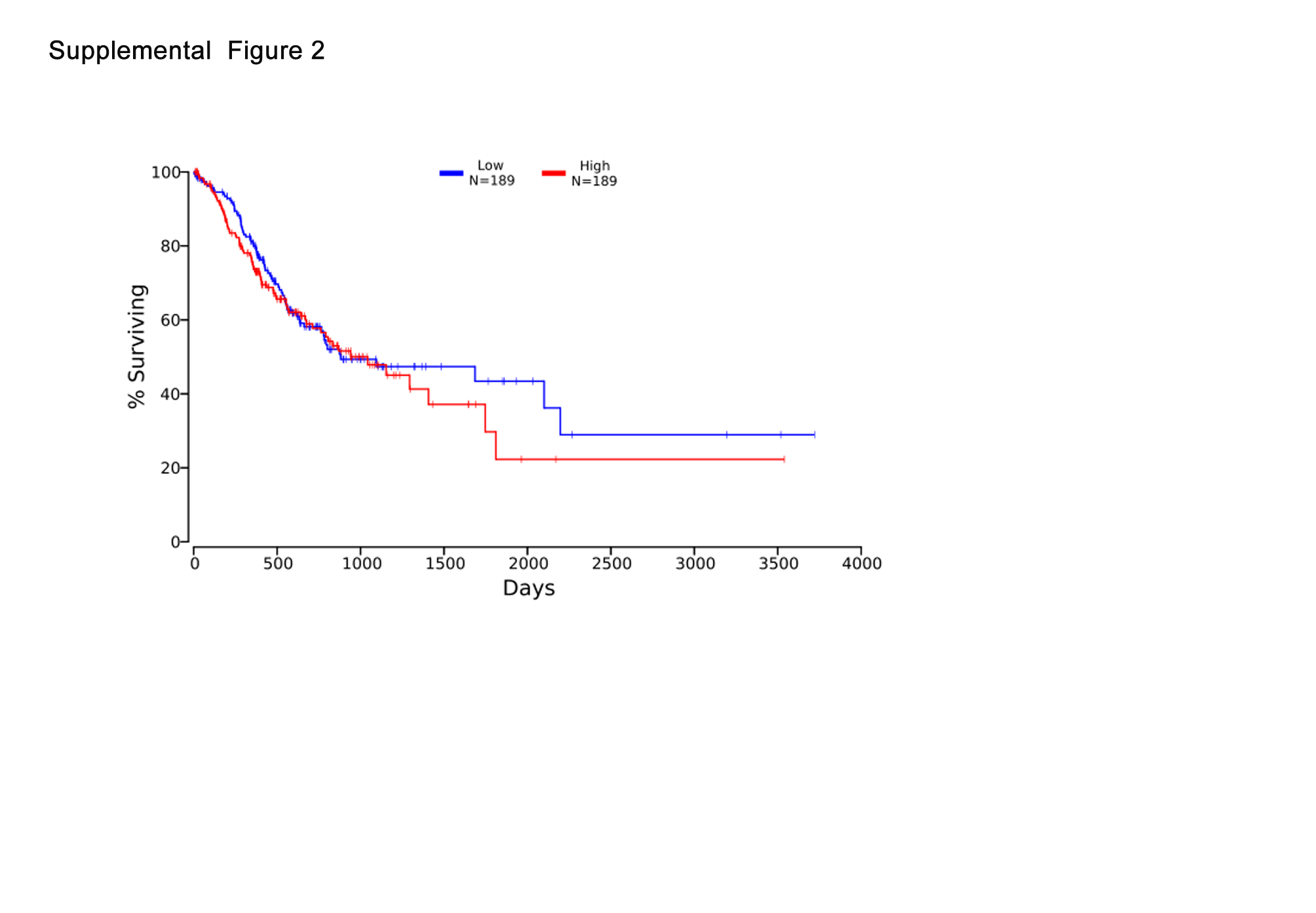

Supplement: Supplementary file 1 — Figure S1. In silico assay of cyclin G2 expression level in gastric cancer from Oncomine. Figure S2. The prognostic value of Cyclin G2 in gastric cancer. Figure S3. Positive and negative controls of immunohistochemistry assay. Bone marrow sections was used to validate cyclin G2 and Ki-67 antibody. Positive immunostaining presented as brown color counterstained with haematoxylin. IgGs against the species where the primary antibody was produced were used as negative controls of the staining (IgG). (ZIP 1323 kb) [file 13046_2018_973_MOESM1_ESM.zip › supplemental figure2.tif]

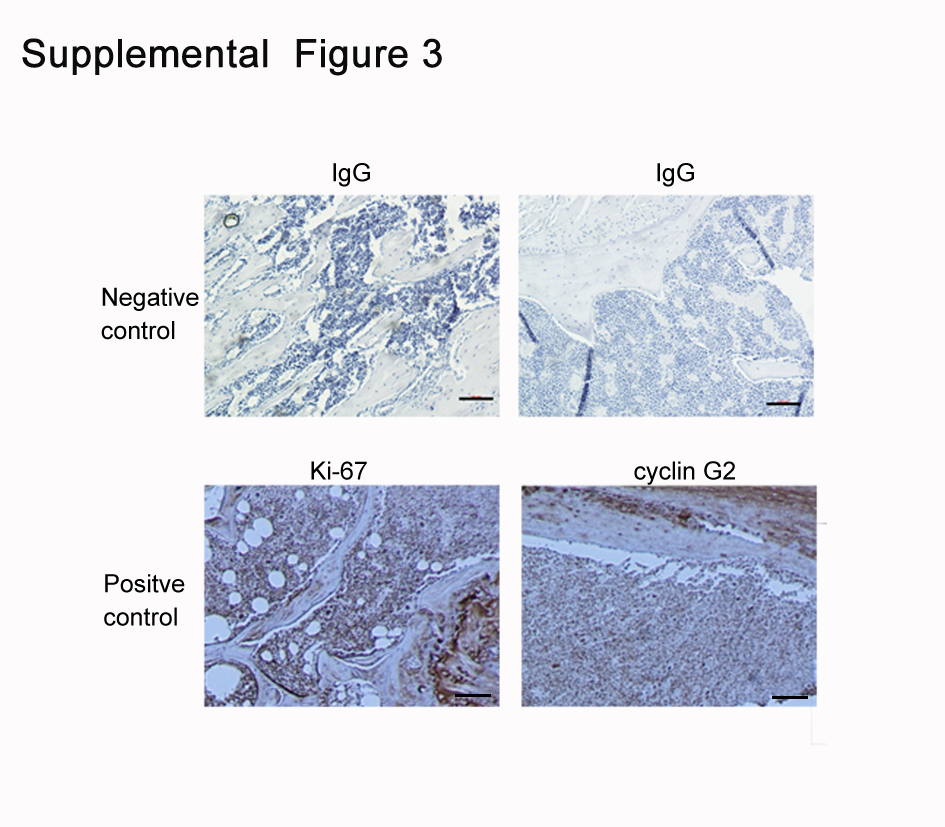

Supplement: Supplementary file 1 — Figure S1. In silico assay of cyclin G2 expression level in gastric cancer from Oncomine. Figure S2. The prognostic value of Cyclin G2 in gastric cancer. Figure S3. Positive and negative controls of immunohistochemistry assay. Bone marrow sections was used to validate cyclin G2 and Ki-67 antibody. Positive immunostaining presented as brown color counterstained with haematoxylin. IgGs against the species where the primary antibody was produced were used as negative controls of the staining (IgG). (ZIP 1323 kb) [file 13046_2018_973_MOESM1_ESM.zip › supplemental figure3.tif]
